# Supplementary material for: Hybrid Models and Biological Model Reduction with PyDSTool
Source: PLoS Comput Biol. 2012 Aug 9;8(8):e1002628. doi: 10.1371/journal.pcbi.1002628 (PMC3415397; doi:10.1371/journal.pcbi.1002628)
Supplement: Text S4 — Complete source code for the PyDSTool package (version 0.88.120504). Includes API documentation and help files linking to web pages. This file is identical to the current public release on Sourceforge.net. (ZIP) [file pcbi.1002628.s004.zip › PyDSTool/html/PyDSTool.MProject.ModelInterface-class.html]

xml version="1.0" encoding="ascii"?


PyDSTool.MProject.ModelInterface


| Home | Trees | Indices | Help | PyDSTool |
| --- | --- | --- | --- | --- |

Package PyDSTool ::
Module MProject ::
Class ModelInterface

[hide private]

[frames] | no frames]

# Class ModelInterface

source code

```
 object --+    
          |    
dsInterface --+
              |
             ModelInterface
```

Known Subclasses:
:   - extModelInterface
    - , intModelInterface

---

```
Model constraints expressed as a uni-directional interface to another
formal system model:
- Made up of conditions imposed on the other system's test trajectory.
- Defines evaluation criteria for any view (e.g. from experimental data and
test conditions).
This is an abstract superclass for the 'internal' and 'external'
sub-classes.
```


|  |  |
| --- | --- |
| Instance Methods | [hide private] |

|  |  |
| --- | --- |
| \_\_init\_\_(self)  x.\_\_init\_\_(...) initializes x; see x.\_\_class\_\_.\_\_doc\_\_ for signature | source code |

|  |  |
| --- | --- |
| \_get\_initiator\_cache(self, ics=None, t0=0)  initiator is a ModelInterface or GeneratorInterface object | source code |

|  |  |
| --- | --- |
| set(self, key, value, ics=None, t0=0) | source code |

|  |  |
| --- | --- |
| get(self, key, ics=None, t0=0) | source code |

|  |  |
| --- | --- |
| Rhs(self, t, xdict, pdict)  Direct access to a generator's Rhs function. | source code |

|  |  |
| --- | --- |
| Jacobian(self, t, xdict, pdict, idict=None)  Direct access to a generator's Jacobian function (if defined). | source code |

|  |  |
| --- | --- |
| JacobianP(self, t, xdict, pdict)  Direct access to a generator's JacobianP function (if defined). | source code |

|  |  |
| --- | --- |
| MassMatrix(self, t, xdict, pdict)  Direct access to a generator's MassMatrix function (if defined). | source code |

|  |  |
| --- | --- |
| AuxVars(self, t, xdict, pdict)  Direct access to a generator's auxiliary variables definition (if defined). | source code |

|  |  |
| --- | --- |
| setup\_conditions(self, conditions, traj) | source code |

|  |  |
| --- | --- |
| evaluate(self, target, force=False)  Evaluate interface consistency against target interface's trajectory on specified conditions. | source code |

|  |  |
| --- | --- |
| \_\_call\_\_(self, target, force=False)  Evaluate interface consistency against target interface's trajectory on specified conditions. | source code |

|  |  |
| --- | --- |
| postprocess\_test\_traj(self, test\_traj)  Called by another interface via get\_test\_traj. | source code |

|  |  |
| --- | --- |
| prepare\_conditions(self, target)  Called automatically by evaluate. | source code |

**Inherited from `dsInterface`**:
`get_test_traj`,
`query`

**Inherited from `object`**:
`__delattr__`,
`__getattribute__`,
`__hash__`,
`__new__`,
`__reduce__`,
`__reduce_ex__`,
`__repr__`,
`__setattr__`,
`__str__`


|  |  |
| --- | --- |
| Class Variables | [hide private] |

\_trajname = `'test_iface_traj'`

**Inherited from `dsInterface`** (private):
`_querykeys`,
`_setkeys`


|  |  |
| --- | --- |
| Properties | [hide private] |

**Inherited from `object`**:
`__class__`


|  |  |
| --- | --- |
| Method Details | [hide private] |

|  |  |
| --- | --- |
| \_\_init\_\_(self)  *(Constructor)* | source code |

x.\_\_init\_\_(...) initializes x; see x.\_\_class\_\_.\_\_doc\_\_ for
signature

Overrides: object.\_\_init\_\_: *(inherited documentation)*

|  |  |
| --- | --- |
| evaluate(self, target, force=False) | source code |

Evaluate interface consistency against target interface's trajectory
on specified conditions.

Optional force argument forces model to recompute its test trajectory,
e.g. because of a known change in model parameters, ics, etc.

|  |  |
| --- | --- |
| \_\_call\_\_(self, target, force=False)  *(Call operator)* | source code |

Evaluate interface consistency against target interface's trajectory
on specified conditions.

Optional force argument forces model to recompute its test trajectory,
e.g. because of a known change in model parameters, ics, etc.

|  |  |
| --- | --- |
| postprocess\_test\_traj(self, test\_traj) | source code |

```
Called by another interface via get_test_traj.
Default post-processing of test trajectory is the identity
 function, i.e. no processing.

Override this method to return a processed version of the
 trajectory or perform other post-computation clean-up, e.g.
 prepare auxiliary feature/condition-related information based
 on end state of trajectory so that HybridModel can use it to
 decide on next hybrid state to switch to.
```

|  |  |
| --- | --- |
| prepare\_conditions(self, target) | source code |

Called automatically by evaluate. Override with user-defined access to
the target interface or processing of trajectory after return of the
target's test trajectory.

  


| Home | Trees | Indices | Help | PyDSTool |
| --- | --- | --- | --- | --- |

|  |  |
| --- | --- |
| Generated by Epydoc 3.0.1 on Fri May 4 15:24:06 2012 | http://epydoc.sourceforge.net |
